# Syntrophic growth via quinone-mediated interspecies electron transfer

**DOI:** 10.3389/fmicb.2015.00121

**Published:** 2015-02-17

**Authors:** Jessica A. Smith, Kelly P. Nevin, Derek R. Lovley

**Affiliations:** Department of Microbiology, University of Massachusetts AmherstAmherst, MA, USA

**Keywords:** *Geobacter*, quinones, AQDS, interspecies electron transfer, syntrophy

## Abstract

The mechanisms by which microbial species exchange electrons are of interest because interspecies electron transfer can expand the metabolic capabilities of microbial communities. Previous studies with the humic substance analog anthraquinone-2,6-disulfonate (AQDS) suggested that quinone-mediated interspecies electron transfer (QUIET) is feasible, but it was not determined if sufficient energy is available from QUIET to support the growth of both species. Furthermore, there have been no previous studies on the mechanisms for the oxidation of anthrahydroquinone-2,6-disulfonate (AHQDS). A co-culture of *Geobacter metallireducens* and *G. sulfurreducens* metabolized ethanol with the reduction of fumarate much faster in the presence of AQDS, and there was an increase in cell protein. *G. sulfurreducens* was more abundant, consistent with *G. sulfurreducens* obtaining electrons from acetate that *G. metallireducens* produced from ethanol, as well as from AHQDS. Co-cultures initiated with a citrate synthase-deficient strain of *G. sulfurreducens* that was unable to use acetate as an electron donor also metabolized ethanol with the reduction of fumarate and cell growth, but acetate accumulated over time. *G. sulfurreducens* and *G. metallireducens* were equally abundant in these co-cultures reflecting the inability of the citrate synthase-deficient strain of *G. sulfurreducens* to metabolize acetate. Evaluation of the mechanisms by which *G. sulfurreducens* accepts electrons from AHQDS demonstrated that a strain deficient in outer-surface *c*-type cytochromes that are required for AQDS reduction was as effective at QUIET as the wild-type strain. Deletion of additional genes previously implicated in extracellular electron transfer also had no impact on QUIET. These results demonstrate that QUIET can yield sufficient energy to support the growth of both syntrophic partners, but that the mechanisms by which electrons are derived from extracellular hydroquinones require further investigation.

## INTRODUCTION

Interspecies electron transfer (IET) may be an important mechanism for energy exchange in a range of anaerobic microbial communities, but the diversity of microbial strategies for IET has yet to be fully explored. The best-known mechanism for IET is hydrogen interspecies electron transfer (HIT) in which the electron-donating species reduces protons to H_2_ and the electron-accepting partner oxidizes H_2_ with the reduction of an electron acceptor (McInerney et al., 2009; Stams and Plugge, 2009; Shrestha and Rotaru, 2014; Sieber et al., 2014). In some instances formate may substitute for H_2_ as the electron carrier [i.e., formate interspecies formate transfer (FIT); McInerney et al., 2009; Stams and Plugge, 2009; Shrestha and Rotaru, 2014; Sieber et al., 2014]. HIT has been documented with a wide diversity of H_2_-producing and H_2_-consuming microbes (McInerney et al., 2009; Stams and Plugge, 2009; Shrestha and Rotaru, 2014; Sieber et al., 2014).

An alternative to HIT and FIT is direct interspecies electron transfer (DIET), in which syntrophic partners forge biological electrical connections to exchange electrons (Summers et al., 2010; Lovley, 2011b; Shrestha et al., 2013a; Rotaru et al., 2014a). To date DIET has only been documented with *Geobacter* species as the electron-donating species with either another *Geobacter* species (Summers et al., 2010; Shrestha et al., 2013a) or a methanogen (Rotaru et al., 2014a,b) as the electron-accepting species. The ability of *Geobacter* species to participate in DIET can be attributed to their ability to make extracellular electrical connections via pili that have metallic-like conductivity (Reguera et al., 2005; Summers et al., 2010; Malvankar et al., 2011; Shrestha et al., 2013a). Conductive materials such as granular activated carbon (Liu et al., 2012), biochar (Chen et al., 2014b), and carbon cloth (Chen et al., 2014a) can substitute for the conductive pili to form interspecies electrical connections. The conductive mineral magnetite can also promote DIET (Kato et al., 2012; Liu et al., 2014) by functioning as an outer-surface *c*-type cytochrome substitute (Liu et al., 2014).

A less explored mechanism of IET is electron exchange via quinone-mediated interspecies electron transfer (QUIET) in which compounds with quinone moieties serve as electron shuttles between the electron-donating and the electron-accepting partner. Quinone moieties are components of humic substances, which are abundant in many soils and sediments and can serve as electron acceptors for microbial respiration (Lovley et al., 1996; Scott et al., 1998; Gralnick and Newman, 2007). The hydroquinones produced can abiotically reduce Fe(III) oxides, regenerating the quinone moieties. The humics-catalyzed electron shuttling between Fe(III) reducing microorganisms and Fe(III) oxides can greatly accelerate the rate of Fe(III) oxide reduction and the degradation of organic contaminants in subsurface sediments by Fe(III) reducers (Lovley et al., 1998). Anthraquinone-2,6-disulfonate (AQDS) can serve as a humic substances analog which microbes can reduce to anthrahydroquinone-2,6-disulfonate (AHQDS). Reduced humics or AHQDS can also serve as an electron donor for microbial reduction of a diversity electron acceptors including: nitrate, selenite, and arsenate (Lovley et al., 1999). Providing AQDS as an electron shuttle promoted IET in cell suspensions of *Geobacter metallireducens* and *Wolinella succinogenes* (Lovley et al., 1999) and in co-cultures of *G. metallireducens* and *G. sulfurreducens* (Liu et al., 2012), but it was not determined in these studies whether either of the partners conserved energy to support growth from QUIET.

Co-cultures of *G. metallireducens* and *G. sulfurreducens* grown in a medium with ethanol as the electron donor and fumarate as the electron acceptor provide a good model system for investigating IET mechanisms because: (1) the two species can only grow in ethanol/fumarate medium if they exchange electrons (Summers et al., 2010); (2) *G. metallireducens* does not produce H_2_ or formate when metabolizing ethanol to acetate, eliminating the possibility of HIT or FIT (Shrestha et al., 2013b); (3) both species can be genetically manipulated (Coppi et al., 2001; Tremblay et al., 2012) facilitating functional studies; and (4) a citrate synthase-deficient mutant of *G. sulfurreducens* which cannot use acetate as an electron donor is available, making it possible to determine if electrons derived from IET can serve as sole electron donor to promote respiration and growth (Shrestha et al., 2013a).

Studies with gene deletion mutants demonstrated that genes for five outer-surface *c*-type cytochromes had to be deleted in order to substantially diminish the ability of *G. sulfurreducens* to reduce humic substances or AQDS (Voordeckers et al., 2010). It is expected that reduced humics and AHQDS are also oxidized at the outer cell surface. Humic substances are too large to enter the cell and like AQDS the size and charge of AHQDS are expected to prevent it from crossing the outer membrane. However, it is unknown what outer-surface proteins may be involved in accepting electrons from reduced humics or AHQDS.

In order to learn more about the potential for QUIET to support growth, and the mechanisms for AHQDS oxidation, studies were carried out with *G. metallireducens/G. sulfurreducens* co-cultures. The results demonstrate that growth of both syntrophic partners via QUIET is possible and that the hypothesized electron acceptors for the oxidation of AHQDS at the outer-surface are proteins different than those involved in AQDS reduction.

## MATERIALS AND METHODS

### LABORATORY STRAINS AND CULTURE CONDITIONS

All *Geobacter* strains were obtained from our laboratory culture collection and routinely cultured under strict anaerobic conditions as previously described (Balch et al., 1979; Coppi et al., 2001). All pure culture strains of *G. metallireducens* were regularly transferred to Fe(III) citrate (FC) medium (Lovley et al., 1993) with 20 mM ethanol provided as the sole electron donor and 56 mM ferric citrate as the sole electron acceptor. All pure culture strains of *G. sulfurreducens* were regularly transferred in donor-free fumarate medium (NBF) (Coppi et al., 2001) with 10 mM acetate provided as the sole electron donor and 40 mM fumarate as the sole electron acceptor. Co-cultures were initiated with equal amounts of both organisms in anaerobic pressure tubes containing 10 mL of NBF medium, with 10 mM ethanol provided as the sole electron donor and 40 mM fumarate as the electron acceptor. Cysteine was omitted from all cultures to eliminate the possibility of a cysteine/cystine electron shuttle between the organisms. DL vitamins were also omitted from the media to eliminate any other possible electron shuttling compounds. Additions of AQDS were made from a concentrated stock to provide a final concentration of 500 μM.

The reduced humics analog, AHQDS, was prepared as previously described (Lovley et al., 1999). Briefly, H_2_ was provided as the reductant in a solution of AQDS in bicarbonate buffer with palladium-coated pellets as the reduction catalyst. The AHQDS was passed anaerobically through a 0.2 μm pore diameter filter into sterile anaerobic pressure tubes containing an atmosphere of N_2_-CO_2_. For AHQDS oxidation experiments *G. sulfurreducens* strains grown in NBF medium with acetate provided as the sole electron donor (NBAF) were transferred (1% inoculum) to 10 mL tubes of NBF medium with AHQDS provided as the sole electron donor to a final concentration of 5 mM.

### ANALYTICAL TECHNIQUES

Organic acids were monitored with high performance liquid chromatography (HPLC) as previously described (Nevin et al., 2008). Changes in ethanol concentration were monitored with gas chromatography as previously described (Morita et al., 2011). AHQDS concentrations were determined by measuring absorbance at 450 nm as described previously (Lovley et al., 1996).

#### Protein determination

Protein concentration was conducted as previously described (Rotaru et al., 2012). Equal volumes of culture were harvested at different time intervals during growth, washed in an isotonic wash buffer, then resuspended in 5% sodium dodecyl sulfate (SDS) and steamed for 5 min. The cell lysate was diluted for protein determination with the bicinchoninic acid assay (Sigma–Aldrich), according to the manufacturer’s instructions.

#### Quantitative PCR

The proportion of *G. sulfurreducens* and *G. metallireducens* cells in co-cultures was determined by quantitative PCR on genomic DNA as previously described (Summers et al., 2010). The *G. metallireducens* specific primer set Gmet_F 5′-ATGGCCCACATCTTCATCTC-3′, Gmet_R 5′-TGCATGTTTTCATCCACGAT-3′, and the *G. sulfurreducens* specific primer set Gsulf_F 5′-CCAGCTACGCCTACTTCTTCTTT-3′, Gsulf_R 5′-AAGCTGTGGTTCAGGAGGTATTT-3′ were used to determine proportions of each species in the co-culture. Genomic DNA was extracted using the Epicenter Master Pure DNA Purification kit (Epicentre Biotechnologies, Madison, WI, USA) following the manufacturer’s instructions.

Power SYBR green PCR master mix (Applied Biosystems, Foster City, CA, USA) and an ABI 7500 real-time PCR system were used to amplify and to quantify the PCR products. Each reaction mixture consisted of forward and reverse primers at a final concentration of 200 nM, 5 ng of gDNA, and 12.5 μl of Power SYBR green PCR master mix (Applied Biosystems).

## RESULTS AND DISCUSSION

### QUIET-BASED GROWTH OF BOTH SYNTROPHIC PARTNERS

In the absence of extracellular electron transport mediators, co-cultures of *G. metallireducens* and *G. sulfurreducens* require ca. 30 days to metabolize ethanol when first initiated, which has been attributed to the time necessary for the co-cultures to initially adapt to produce the biological electrical connections required for DIET (Summers et al., 2010). Faster initial rates of succinate production were observed when AQDS was added to co-cultures as a potential electron shuttle (Liu et al., 2012), but it was not determined whether this QUIET also supported cell growth.

**FIGURE 1:**
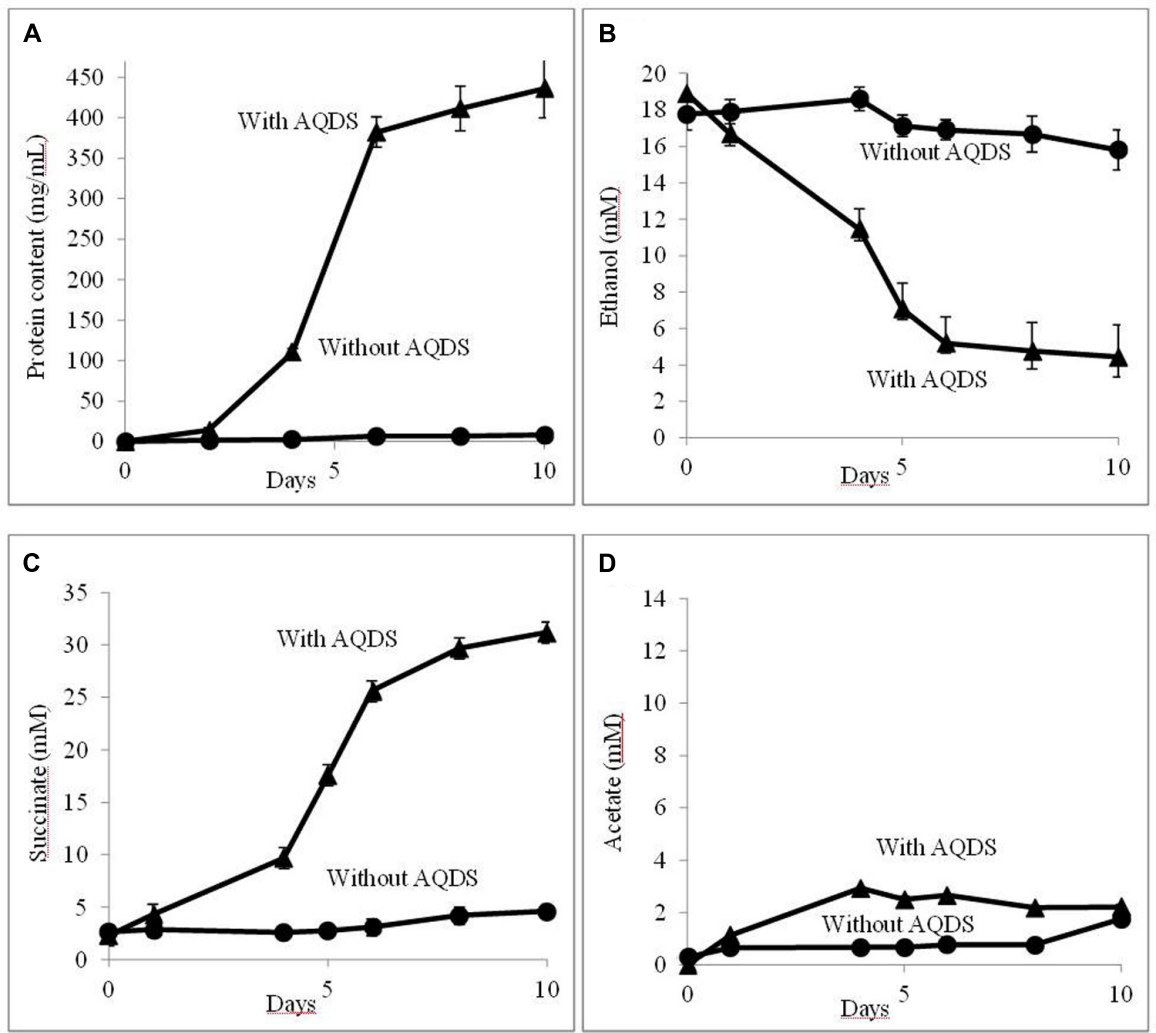
**Growth and metabolism of *Geobacter metallireducens* and *Geobacter sulfurreducens* with and without the addition of AQDS (anthraquinone-2,6-disulfonate) when initially established.** **(A)** cell protein; **(B)** ethanol; **(C)** succinate; and **(D)** acetate over time.

**FIGURE 2:**
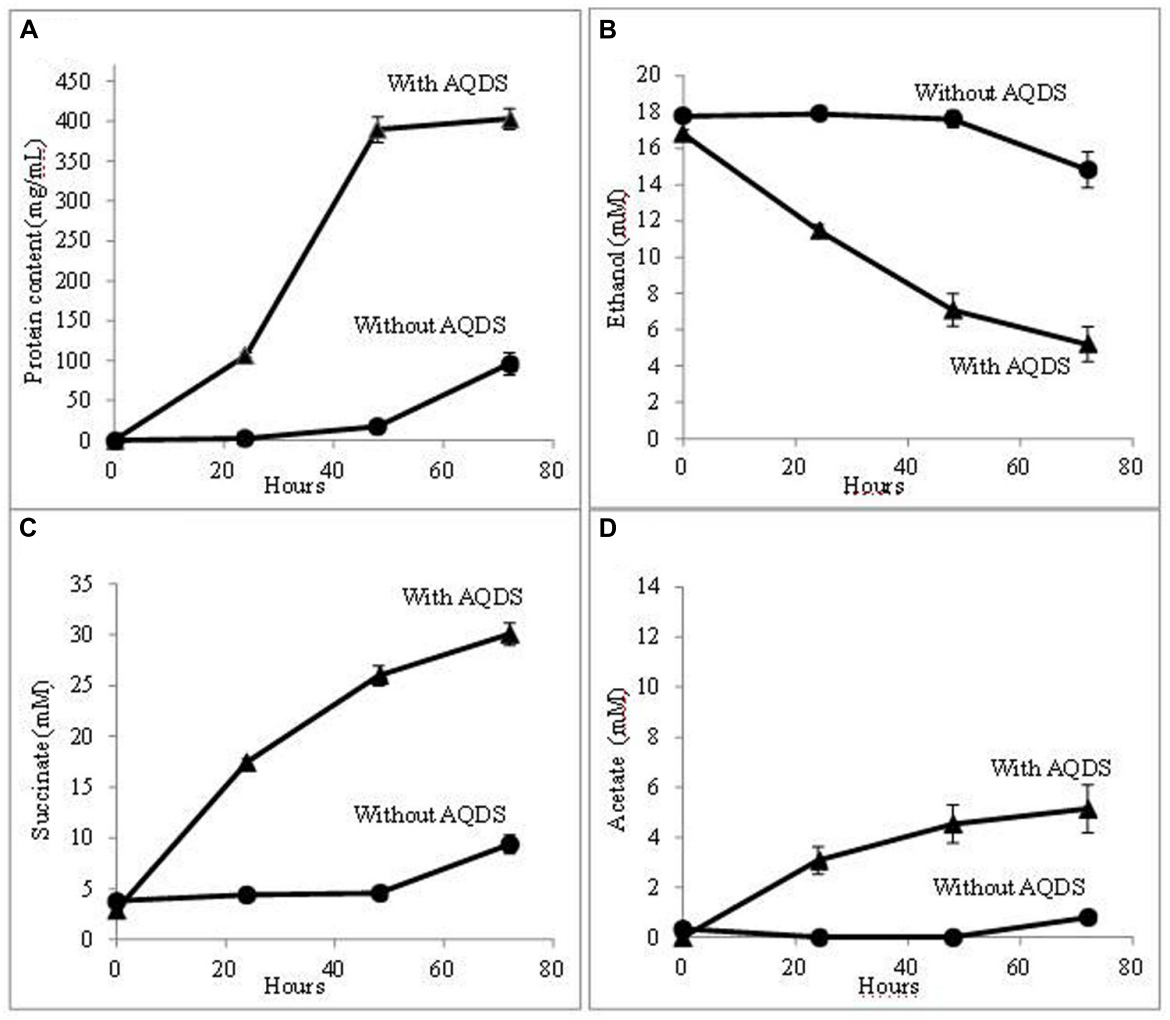
**Growth and metabolism of *G. metallireducens* and *G. sulfurreducens* co-cultures in AQDS-amended medium after four successive transfers.** **(A)** cell protein; **(B)** ethanol; **(C)** succinate; and **(D)** acetate over time.

Further evaluation confirmed that QUIET supported growth of both species (**Figure 1**). Unlike co-cultures participating in DIET, the cells did not aggregate and remained planktonic. The culture medium had an orange tinge characteristic of AHQDS, suggesting that *G. metallireducens* maintained a constant source of AHQDS to serve as an electron donor for *G. sulfurreducens* respiration. Cell protein accumulated over time (**Figure 1A**), coincident with ethanol metabolism (**Figure 1B**) and the reduction of fumarate to succinate (**Figure 1C**). Low levels of acetate accumulated (**Figure 1D**), indicating that *G. sulfurreducens* was consuming most of the acetate that *G. metallireducens* produced from ethanol metabolism. Quantitative PCR revealed that *G. sulfurreducens* accounted for 62 ± 4.5% (mean ± SD; *n* = 3) of the *Geobacter* cells in the co-culture, consistent with *G. sulfurreducens* receiving electrons from the IET via the AQDS/AHQDS electron shuttle, as well as from the acetate released from *G. metallireducens* metabolism of ethanol. With continued transfer of the AQDS-amended co-culture the lag period decreased and the growth rate increased (**Figure 2**), suggesting that the co-culture adapted to optimize QUIET-based growth.

**FIGURE 3:**
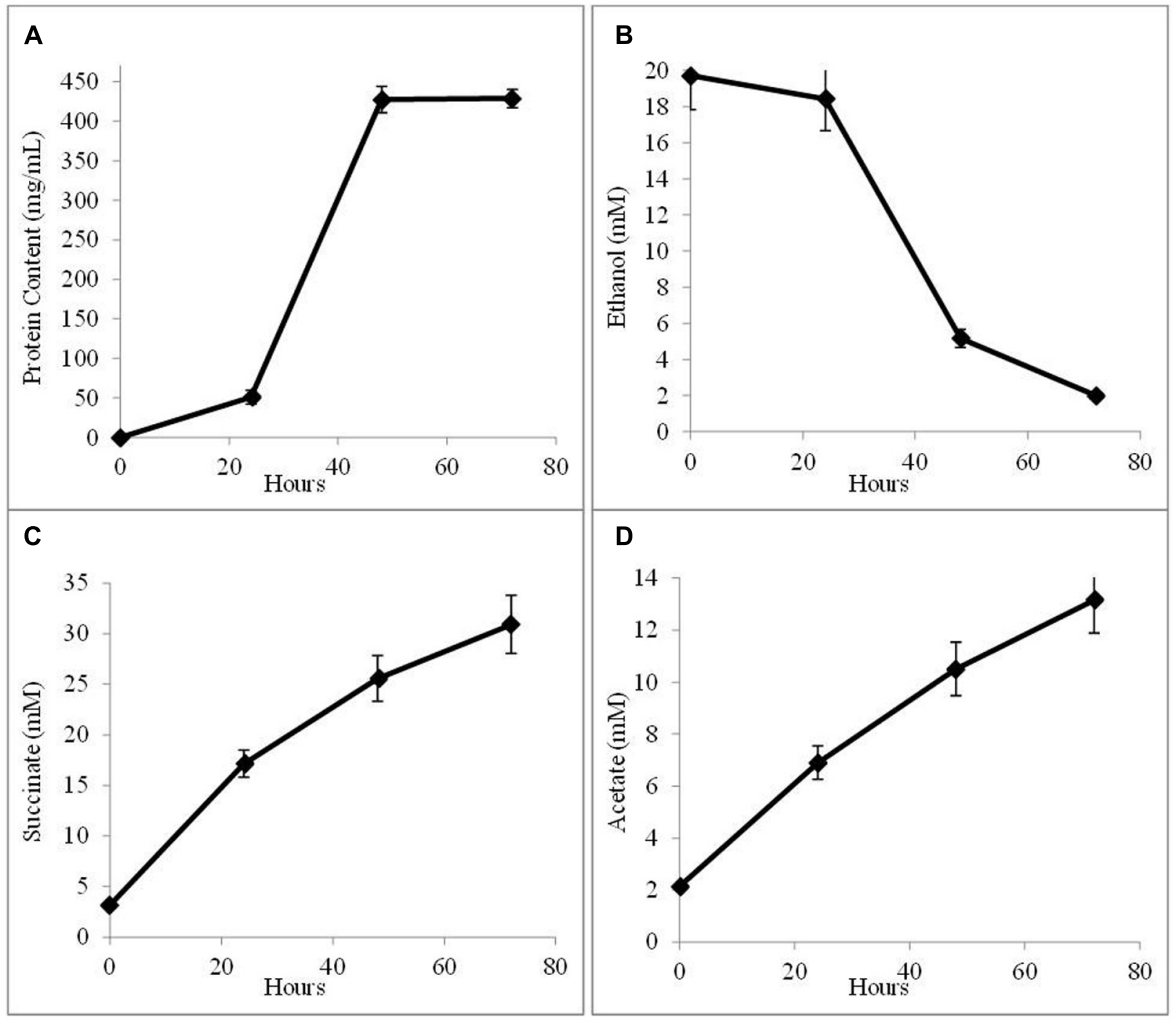
**Growth and metabolism of co-cultures initiated with wild-type *G. metallireducens* and the citrate synthase-deficient strain of *G. sulfurreducens* in AQDS-amended medium after four successive transfers. (A)** cell protein; **(B)** ethanol; **(C)** succinate; and **(D)** acetate over time.

In order to determine whether *G. sulfurreducens* could conserve energy to support growth solely from electrons derived from AHQDS, a co-culture was initiated with the previously described (Ueki and Lovley, 2010) strain of *G. sulfurreducens* in which the gene for citrate synthase was deleted. This strain is unable to use acetate as an electron donor. This co-culture grew, as demonstrated by continued fumarate reduction in successive transfers of a 5% inoculum. Analysis of the fourth transfer of the AQDS-amended *G. metallireducens/G. sulfurreducens* co-culture with the citrate synthase-deficient strain of *G. sulfurreducens* demonstrated that cell protein increased over time (**Figure 3A**), co-incident the ethanol metabolism (**Figure 3B**), and the reduction of fumarate to succinate (**Figure 3C**). There was a steady accumulation of acetate over time (**Figure 3D**), in accordance with the inability of citrate synthase-deficient strain of *G. sulfurreducens* to use acetate as an electron donor. In these co-cultures, the citrate synthase-deficient strain of *G. sulfurreducens* accounted for 51 ± 3.9% of the total cells, comparable to the proportion of *G. sulfurreducens* in the previously described (Shrestha et al., 2013a) co-culture in which *G. metallireducens* and the citrate synthase-deficient *G. sulfurreducens* strain shared electrons via DIET.

**FIGURE 4:**
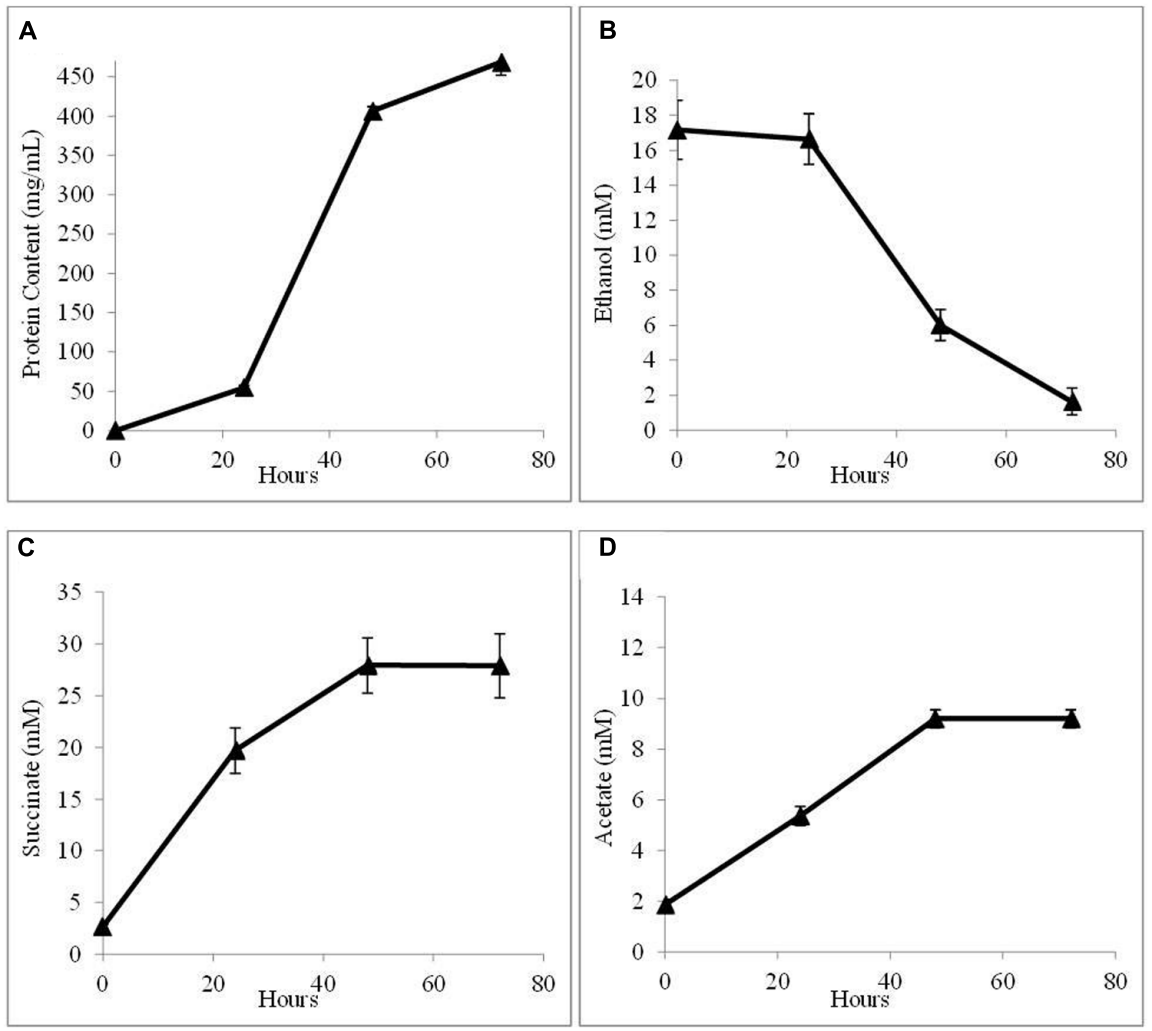
**Growth and metabolism of co-cultures initiated with the pilin-deficient strain of *G. metallireducens* and the citrate synthase-deficient strain of *G. sulfurreducens* in AQDS-amended medium after four successive transfers.** **(A)** cell protein; **(B)** ethanol; **(C)** succinate; and **(D)** acetate over time.

In order to further evaluate the potential for AHQDS to serve as the sole electron donor for *G. sulfurreducens*, a co-culture was initiated with the citrate synthase-deficient strain of *G. sulfurreducens* and the previously described (Tremblay et al., 2012), genetically modified strain of *G. metallireducens* that can not produce the electrically conductive pili required for DIET (Shrestha et al., 2013b). This co-culture grew as well as the co-culture with wild-type *G. metallireducens* (**Figure 4**) with a similar abundance of *G. sulfurreducens* (54 ± 3.7%).

### EVALUATION OF OUTER-SURFACE ELECTRON TRANSPORT COMPONENTS INVOLVEMENT IN AHQDS OXIDATION DURING QUIET

**FIGURE 5:**
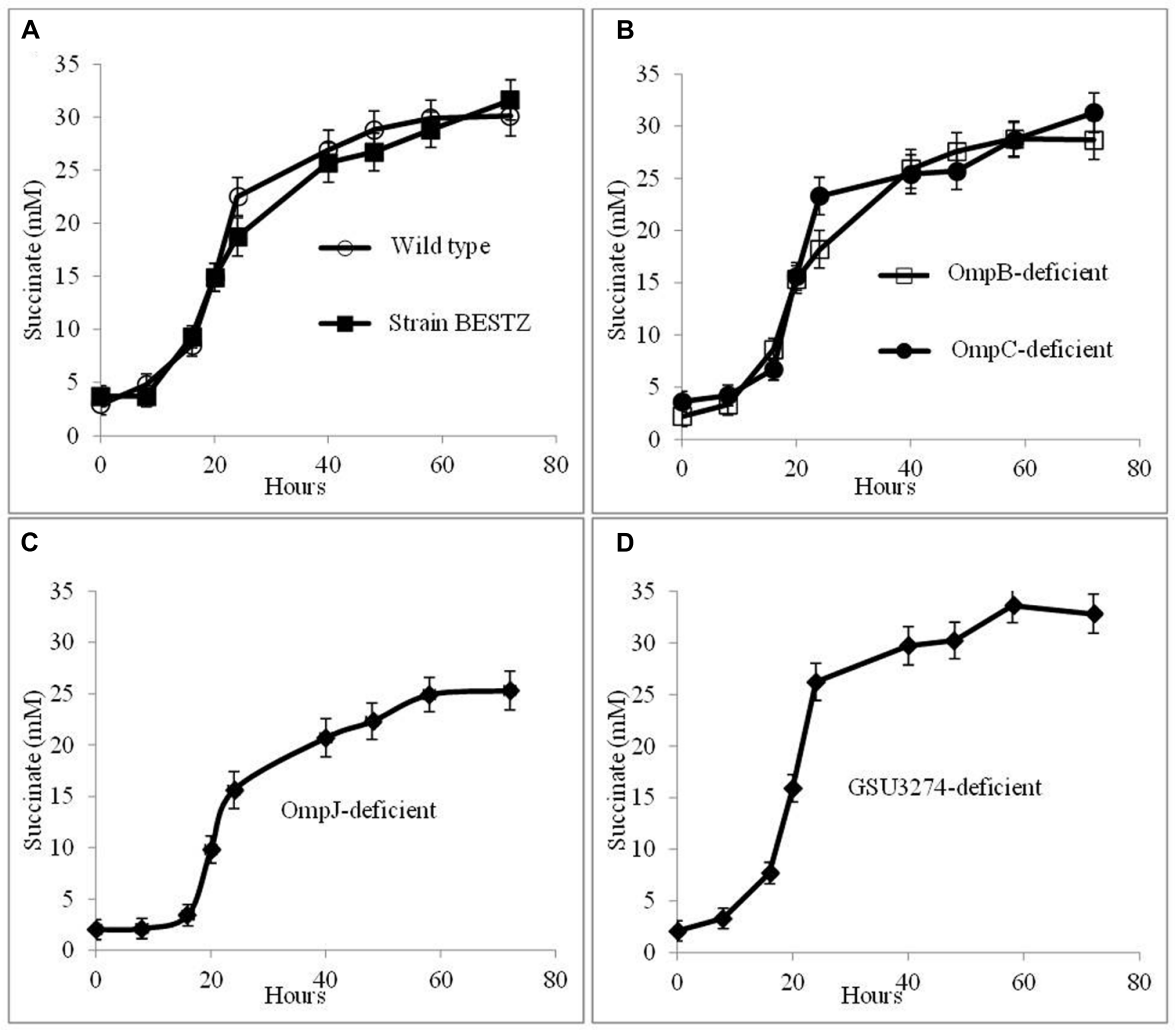
**Growth and metabolism of co-cultures initiated with wild-type *G. metallireducens* and strains of *G. sulfurreducens* in which genes for putative electron transport genes were deleted in AQDS-amended medium after four successive transfers.** *G. sulfurreducens* strains evaluated were: **(A)** wild-type and the BESTZ strain deficient in the outer-surface *c*-type cytochromes OmcB, OmcE, OmcS, OmcT, and OmcA; **(B)** strains deficient in the putative outer-surface multi-copper proteins OmpB of OmpC; **(C)** strain deficient in the putative porin OmpJ; and **(D)** strain deficient in the putative periplasmic *c*-type cytochrome encoded by gene GSU3274.

In order to evaluate potential mechanisms for the hypothesized AHQDS oxidation at the outer cell surface of *G. sulfurreducens*, the impact of deleting genes for outer-surface redox-active proteins on the growth of the co-culture in the presence AQDS was investigated. Co-cultures were initiated with wild-type *G. metallireducens* and the previously described (Voordeckers et al., 2010) BESTZ strain of *G. sulfurreducens* that is deficient in the outer-surface *c-*type cytochromes OmcB, OmcE, OmcS, OmcT, and OmcZ. This strain reduced AQDS at rates less than 5% of the wild-type strain (Voordeckers et al., 2010). However, co-cultures initiated with the BESTZ strain grew with continued transfer in AQDS-amended medium reducing fumarate to succinate at a maximum rate (16.9 ± 1.55 mM succinate produced per day) comparable (**Figure 5A**) to co-cultures initiated with wild-type *G. sulfurreducens* (17.6 ± 1.09 mM succinate produced per day). In contrast, deletion of just *omcS* inhibited DIET in *G. metallireducens/G. sulfurreducens* co-cultures (Summers et al., 2010). Co-cultures in AQDS-amended medium initiated with a strain of *G. sulfurreducens* deficient in only *omcS* also produced succinate at a rate comparable to co-cultures with the wild-type strain (16.7 ± 1.19 mM succinate produced per day). As might be expected, co-cultures in AQDS-amended medium initiated with a strain of *G. sulfurreducens* in which *pilA* had been deleted (Reguera et al., 2005), also functioned well with rates of succinate production of 18.2 ± 1.78 mM succinate produced per day, confirming that conductive pili are not necessary for interspecies electron exchange when AQDS/AHQDS serve as an electron shuttle.

In addition to *c*-type cytochromes, the putative multi-copper, redox-active outer-surface proteins OmpB and OmpC have also been implicated in electron transfer to extracellular electron acceptors (Mehta et al., 2006; Qian et al., 2007; Holmes et al., 2008). However, co-cultures initiated with strains of *G. sulfurreducens* deficient in either OmpB or OmpC effectively reduced fumarate as well as co-cultures initiated with wild-type *G. sulfurreducens* (**Figure 5B**), with rates of succinate production of 17.1 ± 1.4 mM and 17.2 ± 1.4 mM succinate produced per day, respectively.

OmpJ is a putative porin that is one of the most abundant outer-membrane proteins of *G. sulfurreducens* (Afkar et al., 2005). Deletion of the gene encoding for OmpJ in *G. sulfurreducens* reduces the heme content of the cell ca. 50%, particularly reducing the abundance of outer-surface *c-*type cytochromes, but also influencing the relative abundance of cytochromes in other fractions, with increased abundance of some cytochromes and reduced abundance of others (Afkar et al., 2005). The OmpJ-deficient strain reduces fumarate as well as the wild-type, but is deficient in the reduction of soluble FC, as well as insoluble Fe(III) oxide. Yet, co-cultures in AQDS-amended medium initiated with the OmpJ-deficient strain of *G. sulfurreducens* readily reduced fumarate after multiple transfers (**Figure 5C**) with a succinate production rate (15.1 ± 1.01 succinate produced per day) just slightly lower than co-cultures initiated with wild-type *G. sulfurreducens*.

**FIGURE 6:**
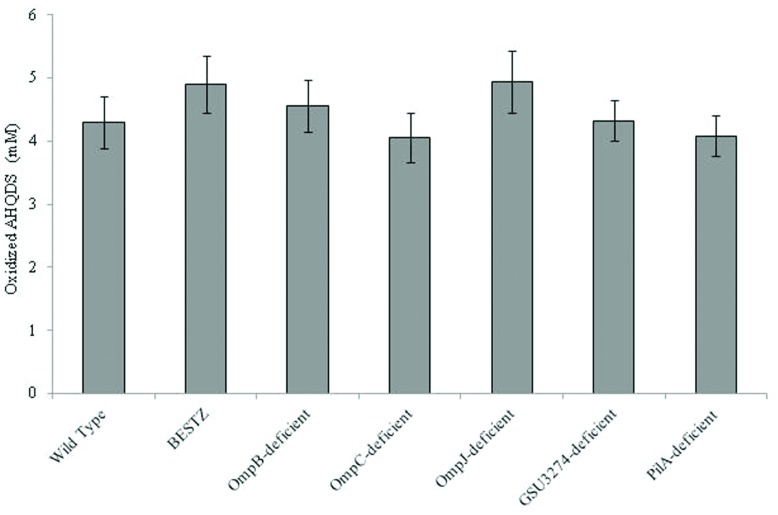
**Quantity of AHQDS (anthrahydroquinone-2,6-disulfonate) oxidized by *G. sulfurreducens* strains grown for 24 h with 5 mM AHQDS as the sole electron donor and 40 mM fumarate as the sole electron acceptor.** The results are the means of triplicate cultures for each strain.

It was surprising that *G. sulfurreducens* did not require outer-surface proteins to function as the electron-accepting partner for QUIET that it requires for the reduction of AQDS or other extracellular electron acceptors. Therefore, in order to further evaluate the ability of the mutant strains to oxidize AHQDS more directly, the strains were inoculated into medium with AHQDS (5 mM) as the sole electron donor and fumarate as the sole electron acceptor. All of the strains tested oxidized AHQDS at rates comparable to wild-type (**Figure 6**).

In addition to extracellular proteins, periplasmic constituents are presumably also required for electron transport from extracellular electron donors to intracellular electron carriers. GSU3274 encodes a putative, periplasmic, mono-heme cytochrome (Strycharz et al., 2011). Wild-type *G. sulfurreducens* can directly accept electrons from a negatively poised graphite electrode for the reduction of fumarate (Gregory et al., 2004), but a strain of *G. sulfurreducens* in which the gene GSU3274 was deleted could not (Strycharz et al., 2011), suggesting that the protein encoded by GSU3274 was an important intermediary in electron transfer from extracellular electron donors. However, AQDS-amended co-cultures initiated with the GSU3274-deficient strain of *G. sulfurreducens* reduced fumarate to succinate as well as co-cultures initiated with the wild-type strain (**Figure 5D**) with a rate of succinate production of 21.6 ± 1.8 mM succinate produced per day. Furthermore, the GSU3274-deficient mutant strain oxidized AHQDS as well as the wild-type in AHQDS/fumarate medium (**Figure 6**).

### IMPLICATIONS

The results demonstrate for the first time that syntrophic partners can conserve energy to support growth via QUIET. This is an important finding because humic substances, which contain abundant quinone moieties that can be reversibly oxidized and reduced, may be important electron carriers in a diversity of anoxic soils and sediments (Gralnick and Newman, 2007). Previously, the primary focus has been on the role of humic substances as an electron shuttle from microorganisms to insoluble Fe(III) oxides, but the results presented here suggest that quinone-bearing organics such as humic substances may also promote IET.

Reduced humic substances clearly must be extracellular electron donors because their size precludes crossing the outer membrane. It was hypothesized that AHQDS is oxidized on the outer cell surface as well because: (1) gene deletion studies have demonstrated that AQDS is reduced on the outer cell surface of *G. sulfurreducens* (Voordeckers et al., 2010) as well as in *Shewanella oneidensis* (Gralnick and Newman, 2007) and (2) the reduced form of the molecule is expected to be excluded from crossing the outer membrane due to similar size and charge considerations. However, none of the outer-surface proteins that have been identified as being important in electron transfer to extracellular electron acceptors were essential for the reverse reaction of oxidizing AHQDS. Further evidence that the route(s) for AHQDS oxidation may differ from those for AQDS reduction is the previous finding that *Paracoccus denitrificans* was incapable of reducing AQDS, but oxidized AHQDS with nitrate as the electron acceptor (Lovley et al., 1999). A better understanding of the mechanisms by which *G. sulfurreducens* oxidizes AHQDS and humic substances could aid in screening the microbial world for other microorganisms that may be capable of functioning as the electron-accepting microorganism in QUIET and may provide insight into the mechanisms for practical applications such as bioremediation and microbial electrosynthesis (Lovley, 2011a; Lovley and Nevin, 2013) in which electrodes serve as the electron donor for anaerobic respiration.

## Conflict of Interest Statement

The authors declare that the research was conducted in the absence of any commercial or financial relationships that could be construed as a potential conflict of interest.
